# Ternary Logic Transistors Using Multi‐Stacked 2D Electron Gas Channels in Ultrathin Oxide Heterostructures

**DOI:** 10.1002/advs.202410519

**Published:** 2024-12-16

**Authors:** Ji Hyeon Choi, Tae Jun Seok, Sang June Kim, Kyun Seong Dae, Jae Hyuck Jang, Deok‐Yong Cho, Sang Woon Lee, Tae Joo Park

**Affiliations:** ^1^ Department of Materials Science and Chemical Engineering Hanyang University Ansan 15588 South Korea; ^2^ Electron Microscopy Research Group Korea Basic Science Institute Daejeon 34133 South Korea; ^3^ Department of Physics Jeonbuk National University Jeonju 54896 South Korea; ^4^ Department of Energy Systems Research and Department of Physics Ajou University Suwon 16499 South Korea

**Keywords:** 2D electron gas, atomic layer deposition, multiple threshold voltage, multi‐stacked channel, ternary logic transistor

## Abstract

2D electron gas field‐effect transistors (2DEG‐FETs), employing 2DEG formed at an interface of ultrathin (≈6 nm) Al_2_O_3_/ZnO heterostructure as the active channel, exhibit outstanding drive current (≈215 µA), subthreshold swing (≈132 mV dec^−1^), and field effect mobility (≈49.6 cm^2^ V^−1^ s^−1^) with a high on/off current ratio of ≈10^7^. It is demonstrated that the Al_2_O_3_ upper layer in Al_2_O_3_/ZnO heterostructure acts as the source/drain resistance component during transistor operations, and the applied potential to the 2DEG channel is successfully modulated by Al_2_O_3_ thickness variations so that the threshold voltage (V_th_) is effectively tuned. Remarkably, double‐stacked 2DEG‐FETs consisting of two Al_2_O_3_/ZnO heterostructured 2DEG channels with a single gate exhibit multiple V_th_, enabling a ternary logic state in a single device. By inducing a voltage difference between the stacked channels, a sequential operation of the upper and lower FETs is achieved, successfully realizing a stable ternary logic operation.

## Graphical abstract

The vertically stacked 2D electron gas (2DEG) channels exhibit multiple threshold voltages (V_th_) in a single device due to different voltage drops in the source/drain contact for each channel. The Al_2_O_3_ film thickness variance between the stacked channels modulates the V_th_ discrepancy, enabling ternary logic switching with sequential operation of the upper and lower 2DEG‐FETs.

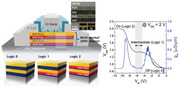

## 1 Introduction

Conventional binary logic system can represent only two states of 0 and 1. However, a multi‐valued logic (MVL) system is capable of articulating three or more states which significantly increases the information processing ability and efficiency in unit device and circuit. This, consequently, leads to a substantial reduction in the number of devices and circuit area required for data processing, thereby, enabling a higher level of device integration feature against an aggressive physical scaling. Since the early 1970s, intensive research have been attempted for the development of circuits beyond binary states‐based operations by a combination of traditional binary devices and circuits to realize multi‐state functionality.^[^ ^1^, ^2^, ^3^ ^]^ However, this circuit‐level approach increased the number of devices and circuit complexity, leading to a low integration density, high power consumption, and slow operation speed. To mitigate these issues, innovative approaches capable of multi‐states operations in a single device are necessary.^[^ ^4^ ^]^

The key challenge in implementing a ternary logic component in a single device lies in achievements of low process complexity, excellent compatibility with existing CMOS processes, and reliable operation of three states in a single device. There have been numerous studies on semiconductor devices exhibiting three or more states based on the multi‐threshold voltage (multi‐V_th_) characteristics. Notable developments include devices utilizing negative differential resistance characteristics realized in the broken band heterojunction structure of 2D transition metal dichalcogenides (TMDCs),^[^ ^5^, ^6^ ^]^ devices incorporating charge confinement by inserting quantum dots or nanocrystals into gate insulator or active layer,^[^ ^7^, ^8^, ^9^ ^]^ and those utilizing heterojunction structures of organic thin films,^[^ ^10^ ^]^ and studies on multi‐state functionality through the modulation of carbon nanotubes’ diameter to adjust V_th_.^[^ ^11^, ^12^ ^]^ These approaches have facilitated the stable implementation of ternary or quaternary states; however, there were limitations in securing reliability and process uniformity due to the difficulty of the fabrication process as well as lack of channel materials.

Meanwhile, a paramount objective of contemporary semiconductor technology is the development of semiconductor bodies with sub‐nanometer thickness. However, several problems in ultrathin body Si leads to significant attention to alternative materials capable of forming an ultrathin body, such as graphene,^[^ ^13^, ^14^ ^]^ 2D TMDCs,^[^ ^15^, ^16^, ^17^ ^]^ and 2D electron gas (2DEG) at the interfaces of semiconductor or oxide heterostructures [e.g., AlGaN/GaN, LaAlO_3_/SrTiO_3_ (LAO/STO)].^[^ ^18^, ^19^, ^20^, ^21^, ^22^ ^]^ While channels based on 2D interfaces or materials offer superior electrical properties compared to Si channels, their suitability for mass production is limited due to the demand for uniformity in the monolayer range and the necessity of single crystalline structures with sharp interfaces. In a pioneering effort, we successfully realized 2DEG at the interface of an oxide heterostructure (3 nm Al_2_O_3_/7 nm TiO_2_) without using single‐crystalline layer, via atomic layer deposition (ALD) process.^[^ ^23^ ^]^

In this study, we demonstrate stacked thin film transistors (TFTs) using 2DEG in an ultrathin (≈6 nm) Al_2_O_3_/ZnO heterostructure fabricated by ALD, exhibiting multiple V_th_ that enable the MVL system. As the source/drain (S/D) electrodes are positioned on the top of the channel (oxide heterostructure), establishing an ohmic contact, the Al_2_O_3_ upper layer serves as a contact resistance (R_C_) between the S/D electrodes and the 2DEG channel, engendering a voltage drop (V_Al2O3_). Given that V_Al2O3_ directly influences the voltage applied to the channel, the manipulation of V_Al2O3_ by varying the Al_2_O_3_ thickness governs V_th_, thereby establishing the inaugural ternary logic system predicated on double‐stacked 2DEG channels in 2DEG‐FETs. The distinct voltage drops across the stacked two 2DEG channels, attributable to varying R_C_, result in distinct V_th_ values for each channel, enabling three switching states.

## 2 Results and Discussion

### 2.1 Electrical Property of 2DEG

**Figure 1:**
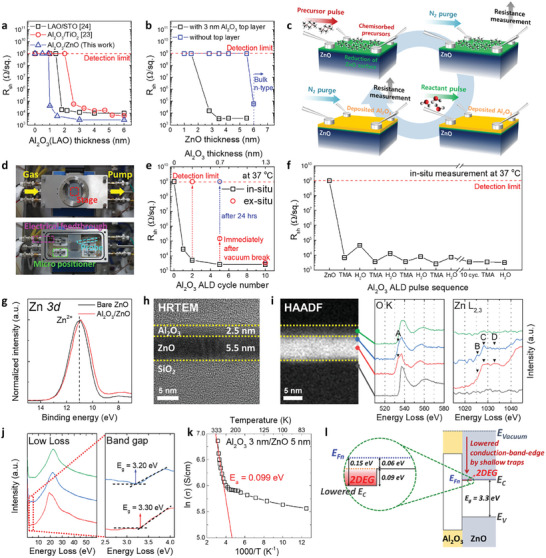
Electrical and physicochemical characteristics in Al_2_O_3_/ZnO 2DEG system. a) R_sh_ of Al_2_O_3_/ZnO heterostructures as a function of Al_2_O_3_ upper layer thickness on 5.5 nm‐thick ZnO lower layer. The results from epitaxial LAO/single crystal STO heterostructures, reproduced with permission,^[^ ^24^ ^]^ Copyright 2010, Springer Nature, and our previous work of ALD‐grown Al_2_O_3_/TiO_2_ heterostructures, reproduced with permission,^[^ ^23^ ^]^ Copyright 2018, American Chemical Society were also included for comparison. The R_sh_ decreased drastically at a specific thickness of each upper layer, implying the typical metal‐insulator transition in 2DEG system. b) R_sh_ of Al_2_O_3_/ZnO heterostructures as a function of ZnO lower layer thickness before and after Al_2_O_3_ deposition. c) A schematic of an in situ ALD process. d) An image of a specially designed in situ ALD reactor with integrated probe tips, enabling simultaneous electrical measurement during ALD process. e) R_sh_ of Al_2_O_3_/ZnO heterostructure measured in the in situ ALD reactor for every ALD Al_2_O_3_ full cycle; (precursor‐purge‐reactant‐purge) and f) for every ALD Al_2_O_3_ half cycle; (precursor‐purge)/(reactant‐purge). g) Normalized Zn 3*d* core‐level XPS spectra of bare ZnO and Al_2_O_3_/ZnO heterostructure. The spectra of Al_2_O_3_/ZnO exhibited a shift toward lower binding energy, indicating that reduction reaction occurs in ZnO during Al_2_O_3_ deposition. h) Cross‐sectional HRTEM image of Al_2_O_3_/ZnO heterostructure and i) corresponding HAADF image with EELS core‐loss spectra of O *K*‐edge and Zn *L_2,3_*‐edge at various positions along the direction perpendicular to the interface. j) EELS low‐loss spectra with estimated band gap at ZnO film (red line) and ZnO surface (blue line). k) Conductivity of Al_2_O_3_/ZnO heterostructure as a function of measurement temperature with calculated V_o_ donor level. l) Electronic band structure of Al_2_O_3_/ZnO heterostructure, showing generated electrons are confined to form 2DEG in quantum well, which is resulted from conduction‐band‐edge lowering effect due to overlapping of V_o_ donor states at the interface.

**Figure** **1**a depicts the sheet resistance (R_sh_) as a function of upper layer thickness in Al_2_O_3_/ZnO heterostructures with a comparison to previous reports (LAO/STO and Al_2_O_3_/TiO_2_),^[^ ^23^, ^24^ ^]^ assessed through Hall measurements in a Van der Pauw configuration. The LAO/STO heterostructure, which was reported by Park et al., was fabricated by depositing an epitaxial STO lower layer on a Si wafer using molecular beam epitaxy and subsequently depositing an epitaxial LAO upper layer using pulsed layer deposition. The Al_2_O_3_/TiO_2_ from our previous report and the Al_2_O_3_/ZnO heterostructures in this study were fabricated on a SiO_2_ (300 nm)/Si substrate by depositing all upper and lower layers using ALD. The thickness of each lower layer in those oxide heterostructures was optimized for creating 2DEG; 100 nm for SrTiO_3_, 8 nm for TiO_2_, and 5.5 nm for ZnO layers. Like the epitaxial interface of LAO/STO and non‐epitaxial interface of ALD‐grown Al_2_O_3_/TiO_2_ heterostructures, both characterized as 2DEG systems, the ALD‐grown Al_2_O_3_/ZnO heterostructures similarly exhibited an abrupt decrease of R_sh_ from >10^9^ Ω sq^−1^ to ≈10^3^ Ω sq^−1^ at a specific Al_2_O_3_ upper layer thickness. This is because the surface of the ZnO lower layer is reduced by highly reducing precursor, trimethylaluminum (TMA) during Al_2_O_3_ deposition, leading to the formation of substantial oxygen vacancies (V_o_) and release of high concentration of electrons. The saturated R_sh_ of Al_2_O_3_/ZnO 2DEG system was approximately halved than that of Al_2_O_3_/TiO_2_ 2DEG system, which can be attributed to the higher intrinsic mobility of ZnO (≈205 cm^2^ V^−1^ s^−1^) than that of TiO_2_ (≈20 cm^2^ V^−1^ s^−1^).^[^ ^25^, ^26^ ^]^ The thickness of the upper layer at which the transition from high to low resistance state occurs is defined as the critical thickness, wherein only an upper layer exceeding this threshold can prevent the oxidation of V_o_, generated by the surface reduction reaction of the lower layer during upper layer ALD, thereby averting 2DEG loss.^[^ ^27^ ^]^ In the Al_2_O_3_/ZnO heterostructure of this study, the critical upper layer thickness is 1–1.5 nm, lower than that of the Al_2_O_3_/TiO_2_ 2DEG system.^[^ ^23^ ^]^ This is because the higher oxidation energy of ZnO than TiO_2_ suppresses the reoxidation of the reduced ZnO surface,^[^ ^28^ ^]^ sustaining a highly conductive 2DEG even with a thinner upper layer.

To demonstrate that high conductivity is not an inherent characteristic of the lower layer but a result of the 2DEG features formed at the interface, the R_sh_ was measured before and after Al_2_O_3_ deposition (3 nm) at different thicknesses of ZnO lower layer as shown in Figure 1b. Prior to Al_2_O_3_ deposition, (blue line) the R_sh_ of the ZnO below a thickness of 5.5 nm showed no conductivity, while for above 6 nm, n‐type conductivity was observed. To exclude the intrinsic attributes of the ZnO lower layer, the upper limit of the thickness allowing for creating 2DEG was set at 5.5 nm. After Al_2_O_3_ deposition (black line), a high R_sh_ (>10^9^ Ω sq^−1^) corresponding to the detection limit was observed for ZnO below 1.5 nm, however, a typical 2DEG system was observed in the range of 3–5.5 nm, where the R_sh_ rapidly decreased to ≈10^3^ Ω sq^−1^. Therefore, the experimentally determined optimal thickness range for the ZnO lower layer to facilitate 2DEG formation is 3–5.5 nm, approximately half that required for the conventional Al_2_O_3_/TiO_2_ 2DEG system (7–10 nm). The sheet carrier density and Hall mobility for the 2DEG within the optimized ZnO thickness range are presented in Figure S1 (Supporting Information).

### 2.2 2DEG Formation Mechanism

To figure out the 2DEG formation mechanism, the change in R_sh_ of Al_2_O_3_/ZnO heterostructure during ALD of Al_2_O_3_ upper layer is monitored in real‐time using a specially designed ALD reactor equipped with the electric feed‐through and oxidation‐resistant probes, which enables in situ resistance measurement during ALD process. Figure 1c,d represent a schematic of the in situ resistance measurement methodology for each pulse of Al_2_O_3_ ALD on ZnO/SiO_2_ substrate and a photograph of the in situ ALD reactor, respectively. The R_sh_ is simultaneously measured for each Al_2_O_3_ ALD cycle or for each precursor and reactant pulse (half‐cycle) on ZnO/SiO_2_ substrate. Figure 1e displays the in situ measured R_sh_ for each ALD cycle of Al_2_O_3_. The R_sh_ sharply decreases to ≈10^4^ Ω sq^−1^ after the first cycle, followed by a further decrease by ≈1 order of magnitude (≈10^3^ Ω sq^−1^) from the second to the fifth cycle. This indicates that substantial surface reduction of ZnO occurred after just one cycle, inducing a significant amount of V_o_ formation and consequently shallow‐level donors, which results in 2DEG formation. The subsequent decrease in R_sh_, attributable to additional surface reduction reaction, reaches a saturation point after five cycles, indicating the completion of 2DEG formation. Given that the establishment of an ALD Al_2_O_3_ monolayer typically occurs within 3–4 cycles, the stabilization of resistance observed beyond this juncture is a reasonable result. To assess the influence of the critical thickness of upper layer on sustaining 2DEG characteristics, the R_sh_ was measured in situ at an upper layer thickness beneath the critical thickness (two cycles), followed by immediate air exposure to gauge the resistance ex situ. The R_sh_ surged to levels beyond measurement capability upon air exposure. An increase in R_sh_ was also observed for an Al_2_O_3_ upper layer slightly thicker yet still under the critical thickness (five cycles) upon air exposure. Although the increase for the five‐cycle scenario was less pronounced, R_sh_ escalated to immeasurable levels after 24 h of air exposure. However, for an Al_2_O_3_ upper layer with 10 cycles (≈1.3 nm), approximating the critical thickness, the low resistance properties were preserved even upon air exposure. This evidence supports the notion that the Al_2_O_3_ upper layer, when exceeding the critical thickness, functions as an oxygen barrier that prevents atmospheric oxygen from infiltrating the heterostructure interface, thereby avoiding oxidation and the loss of V_o_, ultimately maintaining 2DEG conductivity. Consequently, the critical thickness recorded in the ex situ resistance measurements does not relate to the 2DEG formation per se, but represents the minimal thickness required to hinder oxidation to maintain 2DEG by serving as an effective oxygen barrier.

Furthermore, to assess the impact of each pulse in the Al_2_O_3_ ALD process on the formation of 2DEG, which involves the alternating introduction of the highly reducible Al precursor (TMA) and the oxidizing reactant (H_2_O), the R_sh_ was measured for each ALD pulse, as depicted in Figure 1f. The initial TMA pulse led to a swift reduction in R_sh_ to ≈7 × 10^3^ Ω sq^−1^, indicating that the ZnO surface undergoes reduction from the first TMA pulse, initiating 2DEG formation. However, the subsequent introduction of H_2_O increased the R_sh_ by ≈1 order of magnitude, implying that H_2_O reactant oxidizes V_o_ generated by TMA, partially reversing 2DEG formation. In the following cycles, a consistent pattern of R_sh_ decreased during TMA pulses and increased during H_2_O pulses was noted. With progressive cycles, the magnitude of R_sh_ fluctuations gradually lessened, and beyond the critical thickness (10 cycles), H_2_O did not oxidize V_o_ from the 11th TMA/H_2_O pulse onward, stabilizing R_sh_ at ≈3 × 10^3^ Ω sq^−1^. Meanwhile, the deposition of an Al_2_O_3_ upper layer under analogous conditions using dimethylaluminum isopropoxide (DMAIP), a precursor of lesser reducing strength than TMA, consistently manifested high resistance characteristics across all Al_2_O_3_ thicknesses (see Figure S2, Supporting Information). This confirms the significance of surface reduction reactions in 2DEG formation.

### 2.3 Oxygen Vacancy Formation

Physicochemical analyses were performed on the Al_2_O_3_/ZnO heterostructure containing 2DEG. Figure 1g represents the Zn 3*d* core‐level spectra of the bare ZnO (5.5 nm) and Al_2_O_3_ (3 nm)/ZnO (5.5 nm) heterostructure grown on a SiO_2_ substrate, featuring a pronounced peak at ≈11 eV indicative of Zn^2+^ in Zn‐O bonds. After the deposition of the Al_2_O_3_ upper layer, a distinct shift toward lower binding energy was observed, proving the reduction of the ZnO surface during Al_2_O_3_ deposition. The spectra for other elements are included in Figure S3 (Supporting Information).

Figure 1h is a cross‐sectional high‐resolution transmission electron microscopy (HRTEM) image of the Al_2_O_3_/ZnO heterostructure, depicting the amorphous structure of the Al_2_O_3_ upper layer with a thickness of 2.5 nm and the wurtzite crystalline structure of ZnO lower layer with a thickness of 5.5 nm (see Figure S4, Supporting Information). A sharp interface without intermixing between the two films was observed. Figure 1i showcases the results of the STEM‐EELS core‐loss analysis, which substantiated the presence of V_o_ at the interface by examining the defect structure within the Al_2_O_3_/ZnO heterostructure through the analysis of the O *K*‐edge and Zn *L_2,3_*‐edge peaks. The analysis area was divided into four regions: Al_2_O_3_ film (green line), ZnO surface (blue line), ZnO film (red line), and SiO_2_ substrate (black line). The O *K*‐edge peak analysis revealed that the electronic structures of the ZnO and Al_2_O_3_ films (red and green lines) remained unaltered during film growth,^[^ ^29^ ^]^ while the pre‐edge (A peak) intensity selectively diminished on the ZnO surface near the Al_2_O_3_/ZnO interface. This A peak is indicative of orbital hybridization between Zn 3*d*/4*s* state and O 2*p* state,^[^ ^30^, ^31^ ^]^ which implies that the reduced A peak intensity results from decreased orbital hybridization due to structural disorders of V_o_ formation on the ZnO surface during the Al_2_O_3_ deposition. The analysis of the Zn *L_2,3_*‐edge peak revealed electronic structures of the crystalline wurtzite in the ZnO film (red line),^[^ ^32^ ^]^ consistent with the previous HRTEM analysis in Figure S4 (Supporting Information). The pre‐edge (B peak) intensity on the ZnO surface near the Al_2_O_3_/ZnO interface (blue line) increased, alongside variations in the main edge, C and D peaks intensifying and diminishing respectively. The increased B peak intensity at the ZnO surface indicates defect formation and structural distortions, implying structural asymmetry due to chemical reactions on the ZnO surface during Al_2_O_3_ deposition. Peaks C and D reflect the electron transition from Zn 2*p* to mainly unoccupied 4*p* level and the Zn 3*d*‐mixed antibonding state, respectively. An amplified C peak suggests more unoccupied *p* states, while a reduced D peak indicates a partial occupation of the Zn 3*d* antibonding state, which can be interpreted as a signature of reduced Zn‐O orbital hybridization strength (possibly due to V_o_).^[^ ^31^ ^]^

### 2.4 Quantum Well Formation

When defects are introduced within a thin film structure, they modify the electronic band structure, thereby influencing the electrical properties of the film. To study the band structure deformation induced by the formation of V_o_, EELS low‐loss analysis was performed, as illustrated in Figure 1j. Utilizing a monochromator to achieve enhanced energy resolution, spectra within the low energy loss range of ≈1–5 eV were analyzed for the ZnO film (red line) and ZnO surface (blue line), determining the band gap at each location. The band gap measured for the ZnO film was 3.3 eV, whereas it decreased by ≈0.1 eV to 3.2 eV on the ZnO surface. This reduction can be attributed to the overlapping of shallow donor levels created by V_o_ with the ZnO conduction band (CB), consequently lowering the CB minimum (*E_C_*) near the surface of the ZnO film.^[^ ^33^ ^]^ To quantitatively assess the shallow donor level induced by V_o_, conductivity was measured across various temperatures employing cryogenic Hall measurement, with the results depicted in an Arrhenius plot as shown in Figure 1k. A notable increase in conductivity from 80 to 330 K was observed, particularly a significant surge ≈1 order of magnitude near room temperature, indicating that electrons associated with V_o_ are adequately liberated, thus exhibiting highly conductive 2DEG characteristics.

(1) $$\sigma =A\;\exp\;\left(-\mathrm{Ea}/k_{B}T\right)$$

Based on the Arrhenius Equation (1), the linear fitting yielded a slope of −*E_a_*/*k_B_T*, where *σ* denotes the measured conductivity, *A* indicates the Arrhenius constant, *k_B_* denotes the Boltzmann constant, and *T* denotes the absolute temperature. The average donor level (*E_a_*) caused by V_o_ was calculated from the slope (red line) to be ≈0.099 eV below *E_C_*, resulting in the 0.1 eV reduction in the band gap energy observed in the EELS low‐loss analysis results shown in Figure 1j.

Figure 1l schematically represents the electronic band structure of the 2DEG system. The band structure for the ZnO film within the Al_2_O_3_/ZnO heterostructure was reconstructed to differentiate between the surface and bulk of the ZnO film. Assuming the measured carrier density in the Al_2_O_3_/ZnO structure (n_surface_ = ≈10^14^ cm^−2^) as the surface carrier density of ZnO, and that of ≈6 nm‐thick standalone ZnO film beginning to conduct on its own (n_bulk_ = ≈10^13^ cm^−2^) as the bulk carrier density of the ZnO (see Figure S1, Supporting Information), the gap between *E_C_* and *E_F_* of ZnO in each region (surface and bulk) was calculated according to Equations (2) and (3).

(2) $$E_{C,\mathrm{surface}}-E_{F}=k_{B}T\;\ln\;\left(N_{C}/n_{\mathrm{surface}}\right)$$

(3) $$E_{C,\mathrm{bulk}}-E_{F}=k_{B}T\;\ln\;\left(N_{C}/n_{\mathrm{bulk}}\right)$$

where *N_C_* represents the effective density of state at ZnO *E_C_*, calculated as an intrinsic value of the ZnO film itself based on the following equation.

(4) $$N_{C}=2{\left(2\pi m_{e}^{*}\;k_{B}T/h^{2}\right)}^{3/2}$$

From Equation (4), *m_e_* ^*^ is the reported effective mass for ZnO film (0.24 *m_e_*), *h* is the Planck constant, and *N_C_* for ZnO was calculated to be 2.94 × 10^18^ cm^−3^.^[^ ^34^ ^]^ Upon substituting the *N_C_* value and the measured carrier density into Equations (2) and (3), the *E_C,surface_‐E_F_* is −0.15 eV and *E_C,bulk_‐E_F_* is −0.06 eV, as illustrated in Figure 1l. Therefore, an *E_C_* lowering of 0.09 eV is observed near the surface, which is consistent with the analysis results discussed earlier. Consequently, we theoretically and experimentally confirmed the formation of a quantum well on the ZnO surface owing to the *E_C_* lowering effect, where the high‐density electrons released from V_o_ are confined and thus creates 2DEG.

### 2.5 Threshold Voltage Control in 2DEG‐FET

**Figure 2:**
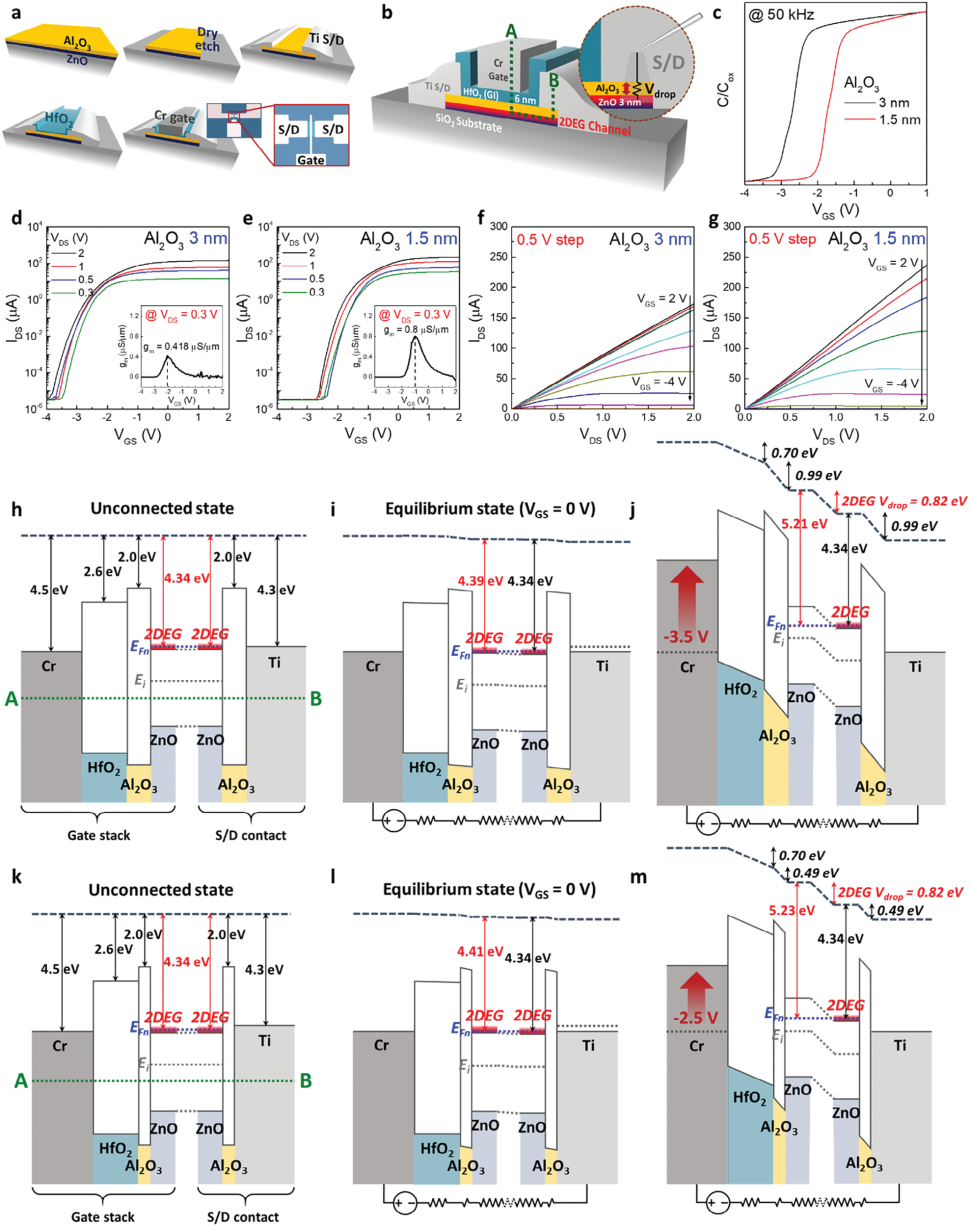
Operation mechanism of 2DEG‐FET. a) Fabrication procedure and b) a schematic of a top‐gated single‐stacked 2DEG‐FET using an ultrathin Al_2_O_3_/ZnO heterostructure. c) Normalized C‐V curves for single‐stacked 2DEG‐FETs with different Al_2_O_3_ layer thickness; 3 and 1.5 nm. Positive shift ≈1 V appears for thinner Al_2_O_3_ layer, indicating |1 V| less V_GS_ is required to make 2DEG channel off. d) Transfer curves of single‐stacked 2DEG‐FETs with 3 nm‐thick and e) 1.5 nm‐thick Al_2_O_3_ layer. The extracted V_th_ for each device are −2.40 and −1.27 V, consistent with the roughly 1 V shift toward positive observed in the C–V curve for thinner Al_2_O_3_, demonstrating enhanced V_ch_ due to lower V_Al2O3_ in thinner Al_2_O_3_ layer. f) Output curves of single‐stacked 2DEG‐FETs with 3 nm‐thick and g) 1.5 nm‐thick Al_2_O_3_ layer for every 0.5 V step of V_GS_. h) Electronic band structures of initial unconnected state, i) equilibrium state at V_GS_ = 0 V, j) non‐equilibrium state with applied V_GS_, which induces potential drop of 0.82 eV in 2DEG channel for using 3 nm‐thick Al_2_O_3_ layer. k) Electronic band structures of initial unconnected state, l) equilibrium state at V_GS_ = 0 V, m) non‐equilibrium state with applied V_GS_ for using 1.5 nm‐thick Al_2_O_3_ layer, demonstrating |1 V| less V_GS_ is required to make the electrical potential in 2DEG channel same with that for using 3 nm‐thick Al_2_O_3_ layer.

**Figure** **2**a,b show the fabrication process and a schematic of 2DEG‐FET, which utilizes 2DEG as a conducting channel, respectively. It was fabricated in the sequence of depositing 3 nm Al_2_O_3_/3 nm ZnO on a 300 nm SiO_2_ substrate, active isolation through dry etch process, Ti (S/D), 6 nm HfO_2_ (gate insulator), and Cr (top gate) deposition. The optical microscope images in Figure 2a confirmed that the patterns were properly formed. The gate width (W_G_), length (L_G_) and the channel width (W_ch_), length (L_ch_) of the fabricated device measure 30, 4, 30, and 14 µm, respectively. This device is a typical coplanar top‐gate structure with the S/D electrodes positioned on the top of Al_2_O_3_/ZnO heterostructured channel ends, as detailed in the enlarged view of the S/D contact area in Figure 2b. Operating in accumulation mode, these TFTs form ohmic contact between the S/D electrodes and the channel, exhibiting linear current variation at the low voltages (see Figure S5, Supporting Information). Thus, the potential equivalent to the difference between the gate voltage (V_GS_) and drain voltage (V_DS_) is applied to the channel. In this context, the Al_2_O_3_ upper layer, situated between the S/D electrodes and the 2DEG channel, functions as a resistor, thereby engendering a voltage drop (V_Al2O3_) in the S/D contact upon the application of V_GS_ and V_DS_. Thus, modulating Al_2_O_3_ thickness permits the control of V_Al2O3_, and consequently, the potential applied to the 2DEG channel (V_ch_) as expressed in Equation (5), demonstrating the feasibility of controlling the V_th_ of the device.

(5) $$V_{\mathrm{ch}}=V_{\mathrm{GS}}-V_{\mathrm{DS}}-V_{\mathrm{Al}2O3}$$

To demonstrate this experimentally, two devices with 3 nm‐ and 1.5 nm‐thick Al_2_O_3_ upper layers (3 nm 2DEG‐FET and 1.5 nm 2DEG‐FET) were fabricated and their electrical properties were compared. Figure 2c displays the capacitance–voltage (C–V) curve measured using the gate and S/D electrodes to ascertain the operational efficacy of the 2DEG as a channel within both devices, with the measurement frequency set at 50 kHz. The outcomes substantiated that for both devices, the 2DEG channels operated as typical semiconductors, showing a decrease in capacitance due to electron depletion caused by a negative V_GS_. Notably, the C–V curve of the 1.5 nm 2DEG‐FET shifted ≈1 V toward the positive compared to the 3 nm 2DEG‐FET, indicating depletion of the 2DEG channel at the lower V_GS_. This evidences that reducing Al_2_O_3_ thickness decreases the V_Al2O3_, thereby increasing V_ch_.

Figure 2d shows the transfer curve of the 3 nm 2DEG‐FET. Applying a positive V_GS_, a conductive 2DEG channel was maintained, exhibiting a high drain current (I_DS_) of ≈140 µA at V_DS_ = 2 V. Conversely, applying a negative V_GS_ depletes electrons in the 2DEG channel, resulting in a decreased current and a complete off state at ≈−3.5 to −4 V. The extremely thin (≈3 nm) ZnO bottom layer ensures the thorough depletion of carriers in the channel, securing an extremely low off current (I_off_) of ≈10^−6^ µA. It should be noted that unlike typical TFTs where both I_on_ and I_off_ are influenced by the channel thickness,^[^ ^35^, ^36^ ^]^ I_on_ in 2DEG‐FET remains unaffected even with an exceedingly thin heterostructure because it depends on the conductivity of the interface 2DEG (see Figure S6, Supporting Information). Consequently, at room temperature, an exemplary on/off current ratio of ≈10^7^ and a commendable subthreshold swing (SS ≈150 mV dec^−1^) were attained. Furthermore, the hysteresis was negligible, and high stability was observed under both positive and negative bias stress, indicating that electron trapping hardly occur within the operating voltage range (see Figures S7 and S8, Supporting Information). The V_th_ was determined based on Equation (6) in the triode region, yielding a value of −2.40 V.

(6) $$I_{\mathrm{DS}}=\left(W_{\mathrm{ch}}/L_{\mathrm{ch}}\right)\times \mu_{\mathrm{FE}}\times C_{\mathrm{ox}}\;\left(V_{\mathrm{GS}}-V_{\mathrm{th}}-V_{\mathrm{DS}}/2\right)\times V_{\mathrm{DS}}$$

Figure 2e shows the transfer curve of the 1.5 nm 2DEG‐FET. Similar to the 3 nm 2DEG‐FET, applying a positive V_GS_ resulted in a high I_DS_ of ≈215 µA at V_DS_ = 2 V, with the current gradually decreasing as electrons in the channel are depleted by the application of a negative V_GS_. Notably, the V_th_ was −1.27 V, which is ≈|1 V| lower than that of the 3 nm 2DEG‐FET (see Figure S9, Supporting Information), corroborating the roughly 1 V shift observed in the C–V curve earlier. This indicates that the reduction in V_Al2O3_ owing to the decreased Al_2_O_3_ thickness enhances V_ch_, thereby reducing |V_th_|. Furthermore, the increased V_ch_ facilitates quicker off behavior compared to the 3 nm 2DEG‐FET, resulting in an improved SS (≈132 mV dec^−1^). The insets of Figure 2d,e illustrate the extracted transconductance (g_m_), with g_m,max_ reaching 0.418 µS µm^−1^ and 0.8 µS µm^−1^, respectively. The g_m,max_ approximately doubling in the 1.5 nm 2DEG‐FET supports its sharp switching characteristics relative to the 3 nm 2DEG‐FET. The maximum field effect mobility (µ_FE,max_) calculated based on Equation (7) is ≈34.5 cm^2^ V^−1^ s^−1^ for the 3 nm 2DEG‐FET and ≈49.6 cm^2^ V^−1^ s^−1^ for the 1.5 nm 2DEG‐FET, surpassing previously reported IGZO, ITZO, and ZnO channels as well as other heterojunction structures featuring 2DEG channels.

(7) $$\mu_{\mathrm{FE}}=\left(L_{\mathrm{ch}}/W_{\mathrm{ch}}\right)\;g_{m}\times 1/\left(C_{\mathrm{ox}}\times V_{\mathrm{DS}}\right)$$

To ascertain the variation in R_C_ attributable to the actual thickness of the Al_2_O_3_ upper layer, resistance measurements were conducted for each L_ch_ in both 2DEG‐FETs (see Figure S10, Supporting Information). The R_C_ of the 3 nm Al_2_O_3_ film was 2483.5 Ω for a channel width of 30 µm, whereas that of the 1.5 nm Al_2_O_3_ film was significantly reduced to 275 Ω, indicating a substantial decrease by approximately an order of magnitude attributable to the thickness reduction. The R_sh_ of the 2DEG channel, deduced from the slope for the 1.5 nm 2DEG‐FET, was ≈2.98 × 10^4^ Ω sq^−1^, ≈1.5 times higher than that of the 3 nm 2DEG‐FET (≈2.00 × 10^4^ Ω sq^−1^), which is consistent with the results from Hall measurements shown in Figure 1a.

Figure 2f,g presents the output curves for the 3 and 1.5 nm 2DEG‐FETs, respectively, with V_GS_ varied in 0.5 V decrements. In both devices, the channel pinch‐off phenomenon, indicative of current saturation, is observed under the condition of V_GS_−V_DS_ < V_th_. Despite the 1.5 nm 2DEG‐FET displaying a higher resistance of the 2DEG channel compared to the 3 nm 2DEG‐FET, the I_on_ under the same voltage drive (V_GS_−V_th_) is higher due to the lower R_C_. As depicted in Figure S10 (Supporting Information), R_C_ increasingly overshadows channel resistance with decreasing L_ch_. Consequently, the total resistance of the 1.5 nm 2DEG‐FET is lower than that of the 3 nm 2DEG‐FET for the device dimension (L_ch_ = 14 µm), thereby enhancing I_on_. The electrical characteristics are summarized and compared in **Table** **1**, with gate leakage current data included in Figure S11 (Supporting Information).

**Table 1:** Electrical properties of 3 and 1.5 nm 2DEG‐FETs. (at V_GS_ = 2 V, V_DS_ = 2 V).

	I_on_ [µA]	I_off_ [µA]	I_on_/I_off_ [−]	V_th_ [V]	SS [mV dec^−1^]	g_m_ [µS µm^−1^]	µ_FE_ [cm^2^ V^−1^ s^−1^]
2DEG‐FET with 3 nm Al_2_O_3_	139.7	3.3 × 10^−6^	≈4.2 × 10^7^	−2.40	150.4	0.418	34.50
2DEG‐FET with 1.5 nm Al_2_O_3_	215.0	3.2 × 10^−6^	≈6.7 × 10^7^	−1.27	131.8	0.8	49.64

### 2.6 Electronic Band Structure of 2DEG‐FETs

To theoretically validate the modulation of V_th_ with the Al_2_O_3_ thickness, the electronic band structure of each device was reconstructed. Figure 2h–j illustrates the electronic band structure of the 3 nm 2DEG‐FET along the A‐B green dotted line, encompassing all films of the gate stack, 2DEG channel, and S/D contact within the schematic in Figure 2b, for the unconnected state, the connected equilibrium state (V_GS_ = 0 V), and the nonequilibrium state with applied V_GS_, respectively. In the band structure of the unconnected state (Figure 2h), where the gate and S/D are not electrically connected, the work functions of Cr and Ti metals are considered as 4.5 and 4.3 eV, respectively,^[^ ^37^ ^]^ with the electron affinities of the HfO_2_, Al_2_O_3_, and ZnO films noted as 2.6,^[^ ^38^ ^]^ 2.0,^[^ ^39^ ^]^ and 4.4 eV,^[^ ^40^ ^]^ respectively. Since the *E_F_‐E_C,bulk_* for the ZnO film was calculated to be 0.06 eV in Figure 1l, the work function of the 2DEG is determined to be 4.34 eV, obtained by subtracting 0.06 eV from the electron affinity of the ZnO film (*E_vacuum_−E_C,bulk_* = 4.4 eV). When the gate and S/D are connected in a circuit (V_GS_ = 0 V), (Figure 2i), the *E_F_* between the two electrodes is aligned, and the difference in work function is distributed as electron potential energy across the entire film. The electric field (*E*) applied to each film is inversely proportional to its permittivity, thereby determining the applied potential to each film as shown in Equation (8), where *ε_0_* denotes the vacuum permittivity (8.84 × 10^−12^ F m^−1^), *k* denotes the dielectric constant, d indicates the film thickness, and *σ* represents the charge density. Considering the dielectric constant and thickness of the HfO_2_, Al_2_O_3_, and ZnO thin films,^[^ ^41^, ^42^, ^43^ ^]^ the calculated electron potential energy applied to the 2DEG (@ZnO) is 0.05 eV, rendering the work function of the 2DEG 0.05 eV higher than in the unconnected state, at 4.39 eV.

(8) $$V=E\;d=\sigma \;d/\left(k\cdot \varepsilon_{0}\right)$$

Figure 2j illustrates the electronic band structure with applied V_GS_, where the potential across each film increases due to V_GS_. Consistent with the C–V measurement outcomes, nearly complete charge depletion is observed at −3.5 V (Figure 2c); at this V_GS_, the change in electron potential energy across each film was calculated. The calculated values for HfO_2_, Al_2_O_3_, and 2DEG (@ZnO) are 0.70, 0.99, and 0.82 eV, respectively, elevating the work function of the 2DEG channel beneath the gate stack to 5.21 eV, which is 0.82 eV higher than at V_GS_ = 0 V. Subsequently, inserting the *E_C,surface_‐E_F_* value of 0.72 eV, calculated from (*E_vacuum_*−*E_F_* = 5.21 eV) – (*E_vacuum_*−*E_C,surface_* = *E_vacuum_*−*E_C,bulk_* + *E_C,bulk_*−*E_C,surface_* = 4.4 eV + 0.09 eV = 4.49 eV), into Equation (2) yielded a carrier density of ≈2.76 × 10^6^ cm^−3^, aligning with the typically reported intrinsic carrier density in ZnO.^[^ ^44^ ^]^ This corroborates that the device switches off upon applying −3.5 V to V_GS_, due to the diminished concentration of 2DEG near the gate stack.

Figure 2k–m illustrates the electronic band structures of a 1.5 nm 2DEG‐FET for the unconnected state, the connected equilibrium state (V_GS_ = 0 V), and the non‐equilibrium state with applied V_GS_, respectively. At V_GS_ = 0 V (Figure 2l), the potential energy applied to the 2DEG is 0.07 eV, resulting in a 2DEG work function of 4.41 eV. Note that as the Al_2_O_3_ thickness decreases, V_Al2O3_ diminishes, consequently increasing the potential energy applied to HfO_2_ and the 2DEG (@ZnO) relative to the 3 nm 2DEG‐FET (HfO_2_: 0.04 to 0.054 eV, 2DEG: 0.05 to 0.07 eV). This suggests that the reduction in Al_2_O_3_ thickness leads to an elevated work function for the 2DEG channel and a decreased |V_th_|. Figure 2m depicts the band structure when the 2DEG channel is off, i.e., when the electron potential energy applied to the 2DEG is 0.82 eV, mirroring the scenario in the 3 nm 2DEG‐FET. The calculated required V_GS_ is −2.5 V, indicating that it is |1 V| smaller than that in the 3 nm 2DEG‐FET (−3.5 V), theoretically verifying the reduction in |V_th_| associated with the decrease in Al_2_O_3_ thickness, observed in Figure 2c–e and Figure S9 (Supporting Information). Thus, it has been theoretically and empirically established that the V_th_ can be modulated without compromising the electrical properties of the channel by adjusting the Al_2_O_3_ upper layer thickness.

### 2.7 Ternary Logic Switching in Multi‐Stacked 2DEG‐FETs

**Figure 3:**
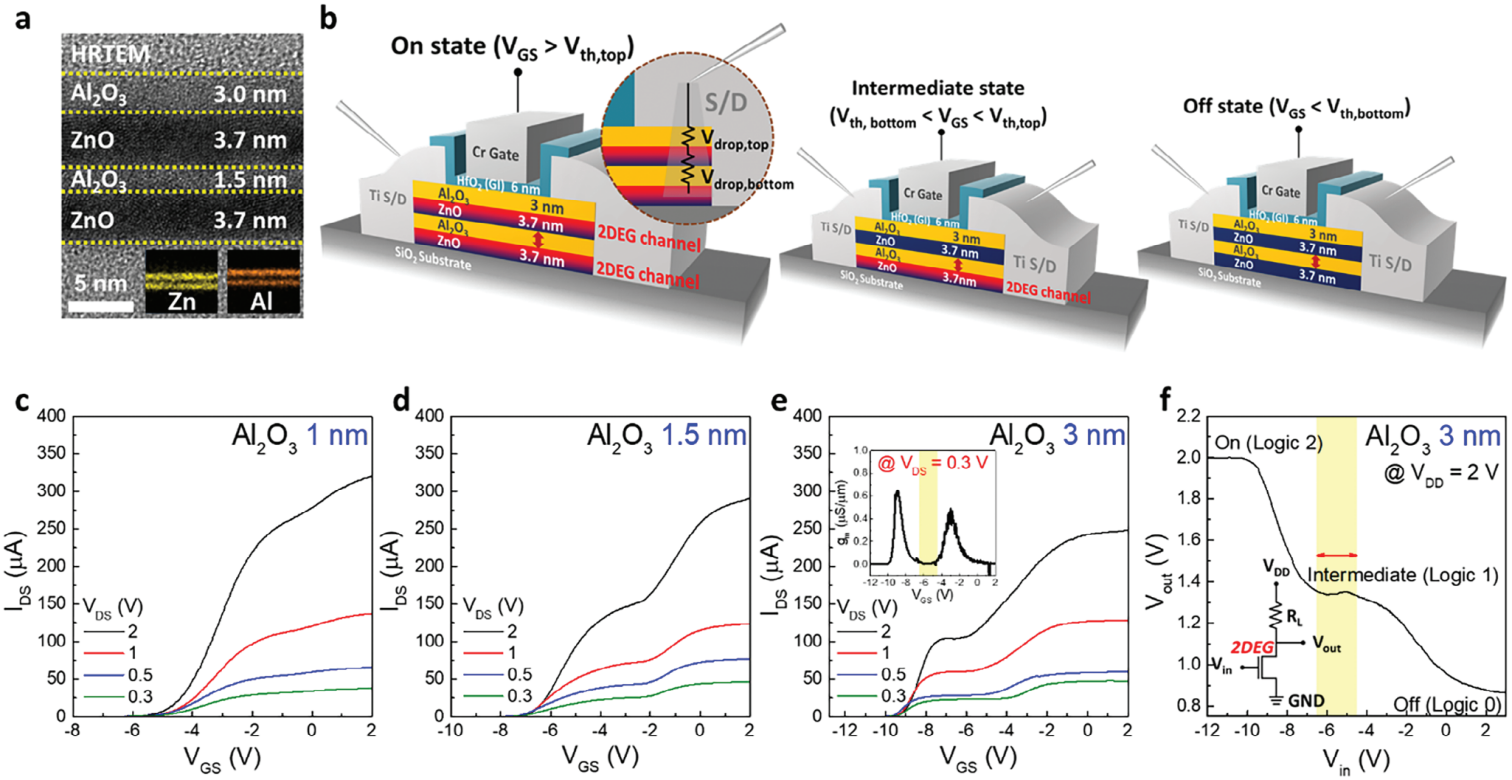
Ternary logic switching characteristics in double‐stacked 2DEG‐FETs. a) Cross‐sectional HRTEM image and EDS mapping of double‐stacked 2DEG heterostructures, displaying clear interface without intermixing layer. b) Schematics of double‐stacked 2DEG‐FETs, indicating three operating modes to realize ternary logic states depending on the V_GS_ range. For on state, both 2DEG channels are all on; for intermediate state, only upper 2DEG channel is off but lower 2DEG channel is still on; for off state, both 2DEG channels are off. c) Transfer curves in linear scale of double‐stacked 2DEG‐FETs with 1 nm‐thick Al_2_O_3_ layer in the lower stack hardly achieve a stable intermediate state. d) Transfer curves with 1.5 nm‐thick Al_2_O_3_ layer in the lower stack show a slight intermediate state, indicating that the upper and lower 2DEG channels start to operate discretely at different V_GS_ range, and therefore, two transfer curves are revealed. e) Transfer curves with 3 nm‐thick Al_2_O_3_ layer in the lower stack display an obvious intermediate state with two distinct transfer curves, indicating each 2DEG channel operates discretely and sequentially at differentV_GS_ range, leading to the realization of ternary logic switching characteristics. The inset shows transconductance curve for V_DS_ = 0.3 V. The yellow box indicates V_GS_ range for the intermediate state; −6.5 to −4.5 V. f) VTC of a resistive‐load ternary NMOS inverter, consisting of double‐stacked 2DEG‐FETs with 3 nm‐thick Al_2_O_3_ layer in the lower stack and an external resistor, resulting in three distinct V_out_; 2, 1.35, 0.86 V. The yellow box indicates V_GS_ range for the intermediate state; −6.5 to −4.5 V. The inset shows the schematic diagram of the circuit for measurement.

Leveraging the V_th_ modulation mechanism, a ternary logic transistor can be realized by vertically stacking two Al_2_O_3_/ZnO heterostructures and modulating voltage drop at S/D contact for each 2DEG channel in a single device. **Figure** **3**a shows the HRTEM image and EDS mapping (inset) of a double‐stacked 2DEG structure fabricated by stacking Al_2_O_3_/ZnO heterostructures. The ZnO lower layers in both the upper and lower stacks are ≈3.7 nm, and the Al_2_O_3_ upper layers in the upper and lower stacks are 3.0 and 1.5 nm, respectively, with a distinct interface without intermixing observed between all thin films. Employing the double‐stacked 2DEG structure as a channel, we fabricated a double‐stacked 2DEG‐FET under a single gate. Figure 3b is a schematic of the device operation. Note that for the lower 2DEG channel, the R_C_ increases because of the thicker film (Al_2_O_3_/ZnO/Al_2_O_3_) between the S/D electrodes and the channel compared to that for the upper 2DEG channel (Al_2_O_3_), resulting in disparate V_th_ values for the upper and lower 2DEG channels with applied V_GS_ (the |V_th_| of the lower 2DEG channel is higher). Consequently, with a given V_GS_, this configuration facilitates three switching logic states: both channels are turned on (on state), the upper channel is turned off while the lower channel is on (intermediate state), and both channels are turned off (off state). For this operation, it should be noted that i) the R_C_ should be remained sufficiently low, necessitating an extremely thin channel thickness, and ii) each channel must be insulated from one another for independent operation. Therefore, the Al_2_O_3_/ZnO heterostructured 2DEG system, which has a thickness of ≈5−6 nm and inherently includes an Al_2_O_3_ insulating layer between the channels, represents an optimal structure for exhibiting MVL characteristics. Furthermore, precise thickness control of Al_2_O_3_ in the lower stack facilitates the adjustment of the V_th_ difference between 2DEG channels.

To explore the mechanism, the Al_2_O_3_ thickness in the upper stack was fixed at 3 nm, while in the lower stack was varied at 1, 1.5, and 3 nm. This approach aimed to maintain the V_th_ of the upper 2DEG channel constant while modulating the V_th_ of the lower 2DEG channel to assess MVL characteristics. Even though the minimal Al_2_O_3_ thickness necessary to sustain 2DEG characteristics (critical thickness) was determined to be ≈1.3 nm (Figure 1e), the lower 2DEG could still be preserved even with 1 nm‐thick Al_2_O_3_ upper layer as the ZnO lower layer in the upper stack is deposited immediately without atmospheric exposure. Figure 3c–e represents the transfer curves of devices with varying Al_2_O_3_ thickness in the lower stack (1, 1.5, and 3 nm). The device dimensions are identical to those of a single‐stacked 2DEG‐FET. The I_on_ when both 2DEG channels are turned on reaches ≈320, ≈291, ≈248 µA at V_GS_ = 2 V, V_DS_ = 2 V for 1, 1.5, 3 nm of Al_2_O_3_, respectively. These values are lower than the combined currents of the single‐stacked 2DEG‐FETs with equivalent Al_2_O_3_ upper layer thicknesses (e.g., 291 µA < 140 µA+215 µA, 248 µA < 140 µA+140 µA). This is attributed to the increased effective capacitance equivalent thickness (CET) and R_C_ for the FET with the lower stacked channel. An outstanding I_on_/I_off_ ratio of 10^7^–10^8^ was observed due to an extremely low I_off_ (≈10^−6^ µA), comparable to that of a single‐stacked 2DEG‐FET. Additionally, with the increasing Al_2_O_3_ thickness in the lower stack, the gate controllability over the lower 2DEG channel diminishes, leading to a gradual increase in |V_th_|, and a simultaneous increase in SS. The distinct electrical characteristics are summarized and compared in **Table** **2**, with the transfer curves (log scale), output curves, and gate leakage current for each device included in Figures S12 and S13 (Supporting Information). For the double‐stacked 2DEG‐FET with 1 nm‐thick Al_2_O_3_ (Figure 3c), the minimal V_Al2O3_ yields an inadequate V_th_ difference between the upper and lower 2DEG channels, precluding the establishment of a stable intermediate state. However, for double‐stacked 2DEG‐FETs with 1.5 nm‐ and 3 nm‐thick Al_2_O_3_ (Figure 3d,e), the increased V_Al2O3_ facilitates a sufficient V_th_ difference between the two channels, enabling the occurrence of an intermediate state. This phenomenon was also observed in the C–V characteristics (see Figure S14, Supporting Information). Furthermore, the device‐to‐device variation for double‐stacked 2DEG‐FETs with 3 nm‐thick Al_2_O_3_, integrated on a 4‐inch wafer, was evaluated to be negligible (see Figure S15, Supporting Information).

**Table 2:** Electrical properties of double‐stacked 2DEG‐FETs with 1 nm, 1.5 nm, and 3 nm‐thick Al_2_O_3_ layer in the lower stack (at V_GS_ = 2 V, V_DS_ = 2 V).

	I_on_ [µA]	I_off_ [µA]	I_on_/I_off_ [−]	V_th_ [V]	SS [mV dec^−1^]
Double‐stacked 2DEG‐FET with 1 nm Al_2_O_3_	319.9	4.2 × 10^−6^	≈7.6 × 10^7^	−4.42	147.3
Double‐stacked 2DEG‐FET with 1.5 nm Al_2_O_3_	290.7	3.8 × 10^−6^	≈7.6 × 10^7^	−6.57	163.5
Double‐stacked 2DEG‐FET with 3 nm Al_2_O_3_	248.2	1.9 × 10^−6^	≈1.3 × 10^8^	−9.03	180.0

Figure 3f illustrates the voltage transfer curve (VTC) measured in a resistive‐load ternary NMOS constructed by connecting an external resistor to the double‐stacked 2DEG‐FET with 3 nm‐thick Al_2_O_3_, which exhibits the most distinct intermediate state. The measurement circuit's schematic is depicted in the inset. The input voltage (V_in_) was varied across the same V_GS_ range as the transfer curve, with the external resistor (R_L_) of 18 kΩ and an operating voltage (V_DD_) was 2 V. Due to the sequential operation of 2DEG channels depending on the V_in_, three distinct output voltages (V_out_ = 2, 1.35, 0.86 V) were observed, representing logic “high,” “intermediate,” and “low” states (2, 1, 0), respectively. The V_in_ range corresponding to the intermediate state (yellow box in Figure 3f) coincides with the V_GS_ range in the inset of Figure 3e without g_m_ variation (−6.5 to −4.5 V), validating this range as representative of the intermediate state. The VTC for double‐stacked 2DEG‐FETs with 1 nm‐ and 1.5 nm‐thick Al_2_O_3_ are reflected in Figure S16 (Supporting Information). In respect to inverter operation, since MVL transistors exhibit a smaller noise margin compared to binary transistors, it is crucial to ensure an adequate intermediate state range, which can be further extended by reducing the Al_2_O_3_ thickness in the upper stack (see Figure S17, Supporting Information). Ultimately, by modulating the V_th_ difference between the stacked 2DEG channels, we successfully realized a ternary logic system in a single device based on 2DEG for the first time.

## 3 Conclusion

Highly conductive 2DEG was created at the interface of ultrathin Al_2_O_3_(≈3 nm)/ZnO(≈3 nm) heterostructure. In situ analyses identified that the V_o_ generated by the surface reduction reaction during the Al_2_O_3_ deposition constitutes the mechanism underpinning the 2DEG formation. The 2DEG‐FET in this study exhibited outstanding electrical characteristics compared to several preceding oxide heterojunction channel‐based devices. In particular, by controlling the Al_2_O_3_ thickness which acts as the R_C_ during device operation, V_th_ was successfully modulated without compromising the electrical performance of the channel. The device operation mechanism was demonstrated through the proposed electronic band structure derived from theoretical calculations alongside empirical measurements and analyses. Double‐stacked 2DEG FETs integrating two 2DEG channels were fabricated by vertically stacking Al_2_O_3_/ZnO heterostructures. The V_th_ discrepancy between the upper and lower 2DEG channels was induced by varying Al_2_O_3_ thickness between the stacked 2DEG channels, effectuating ternary logic states. Unlike previously reported MVL transistors, this novel 2DEG MVL transistor is promising for future electronics, as it substantially simplifies the fabrication process while ensuring high compatibility with existing semiconductor processes and materials.

## 4 Experimental Section

### Ultrathin Al_2_O_3_/ZnO Heterostructure

The ZnO thin films were grown by ALD on a thermally‐grown SiO_2_ (300 nm)/Si substrate at 150 °C in a four‐inch traveling‐wave type ALD reactor. Diethylzinc [DEZ, (C_2_H_5_)_2_ Zn] and H_2_O were used as the Zn precursor and oxygen source, respectively. The purge/carrier gas was high‐purity N_2_ (99.999%) with 200 sccm (standard cubic centimeter per minute) flowing horizontally along the reactor. The Al_2_O_3_ thin films were grown by ALD at 300 °C on the ZnO film in sequence where trimethylaluminum [TMA, Al(CH_3_)_3_], dimethylaluminum isopropoxide [DMAIP, (CH_3_)_2_Al(OC_3_H_7_)] and H_2_O were used as the Al precursor and oxygen source, respectively, for the formation of the Al_2_O_3_/ZnO heterostructure. Film thickness was measured using spectro‐scopic ellipsometry (SE, MG‐1000, Nano View Co.) with a 380–900 nm spectral range and a 70° fixed incidence light angle.

### Electrical Measurements and Physicochemical Analysis

In the electrical measurements, the Ti (100 nm) top electrodes were deposited through a shadow mask at four corners of square samples (15 × 15 mm) using electron‐beam evaporator. The R_sh_, carrier density, and mobility were measured using the Hall measurement system (Ecopia Co., HMS‐5500). In situ resistance measurement with four‐point probe was performed using specially designed ALD reactor which was modified based on micro vacuum probe station system equipped with ultra‐high vacuum‐compatible ceramic heater. The electric power of heater was supplied through the nine‐pin electric feedthrough which was separated from signal feedthrough. The surface of probe tip was coated with Au to prevent oxidation during ALD process. In order to perform the measurement, the device was cooled down immediately after the deposition. To shorten the cooling time, a slightly higher measurement temperature of 37 °C was selected, at which the same resistance can be achieved as at room temperature. During the cooling process, inert N_2_ gas was introduced at a flow rate of 200 sccm to further reduce the cooling time and prevent possible oxidation.

The microstructures of the Al_2_O_3_/ZnO heterostructures including crystallinity were observed by HRTEM (JEOL, JEM‐2100F) with SAED. XPS (Thermo Fisher Scientific Co.) analysis was performed to determine the valence states of Zn at the interface of the Al_2_O_3_/ZnO heterostructure. Electronic structures of O *K*‐edge and Zn *L_2,3_*‐edge to analyze defect states were obtained by Monochromated STEM‐EELS with GIF Continuum HR 1066 spectrometer (JEOL, Monochromated JEM‐ARM 200F, DJ105, KBSI).

### Fabrication of Field‐Effect Transistors

For the fabrication of 2DEG FETs, Al_2_O_3_ (≈3 nm)/ZnO (≈3 nm) thin films were grown by ALD at 300 and 150 °C, respectively, on a thermally grown four‐inch SiO_2_ (300 nm)/Si wafer. The Al_2_O_3_/ZnO active layer was patterned using photolithography (MDA‐400 M, MIDAS system) with positive photoresist (AZ GXR‐601 14CP) and developer (AZ 300 MIF). Inductively coupled plasma dry etching (STS Multiplex ICP) was applied for active isolation. All PR residue was removed through O_2_ plasma using asher (PE 1000, advanced energy). S/D was patterned using photolithography with negative photoresist (DNR‐L300‐D1 21.8 CP) and developer. Ti metal (≈100 nm) was deposited by electron‐beam evaporation and patterned by a lift‐off process. Then, a 6 nm thick ALD HfO_2_ film was deposited as a gate insulating film at 250 °C using Tetrakis(ethylmethylamino) Hafnium [TEMAHf, Hf[N(CH_3_)C_2_H_5_]_4_] as a precursor and H_2_O as a reactant. Finally, top gate was patterned with Cr metal (≈100 nm) in the same way as S/D patterning. The W_G_, L_G_ and W_ch_, L_ch_ of all fabricated devices here measure 30, 4, 30, 14 µm, respectively. Typical transfer and output characteristics were measured using a semiconductor parameter analyzer (Keithley 4200A‐SCS) at room temperature. C‐V characteristics were measured using a precision LCR meter (Keysight Co., E4980A) at room temperature.

### Construction of Ternary NMOS Inverters

Ternary logic transistor (double‐stacked 2DEG FETs) was connected with an external resistor of 18 kΩ for construction of resistive‐load ternary NMOS inverter. The source and top gate pads of active transistor were in contact as ground (GND) and V_GS_ (V_in_) terminals, respectively. One side of resistor was in contact as V_DD_ terminal and the other side, which was connected with the drain pad of active transistor, was in contact as V_out_ terminal to measure the VTC. Typical VTC were measured using a semiconductor parameter analyzer (Keithley 4200A‐SCS) at room temperature.

## Conflict of Interest

The authors declare no conflict of interest.

## Supporting information



Supporting Information

## Data Availability

The data that support the findings of this study are available from the corresponding author upon reasonable request.
