# Supplementary material for: Ternary Logic Transistors Using Multi‐Stacked 2D Electron Gas Channels in Ultrathin Oxide Heterostructures
Source: Adv Sci (Weinh). 2024 Dec 16;12(6):2410519. doi: 10.1002/advs.202410519 (PMC11809351; doi:10.1002/advs.202410519)
Supplement: Supplementary file 1 — Supporting Information [file ADVS-12-2410519-s001.pdf]

ADVANCED SCIENCE
Open Access

Supporting Information

for *Adv. Sci.*, DOI 10.1002/advs.202410519

Ternary Logic Transistors Using Multi-Stacked 2D Electron Gas Channels in Ultrathin Oxide Heterostructures

Ji Hyeon Choi, Tae Jun Seok, Sang June Kim, Kyun Seong Dae, Jae Hyuck Jang, Deok-Yong Cho, Sang Woon Lee and Tae Joo Park**

Supporting Information

Ternary Logic Transistors Using Multi-Stacked Two-Dimensional Electron Gas Channels in Ultrathin Oxide Heterostructures

Ji Hyeon Choi, Tae Jun Seok, Sang June Kim, Kyun Seong Dae, Jae Hyuck Jang, Deok-Yong Cho, Sang Woon Lee, and Tae Joo Park**

J. H. Choi, T. J. Seok, S. J. Kim, T. J. Park
Department of Materials Science and Chemical Engineering, Hanyang University, Ansan 15588, Korea
E-mail: tjp@hanyang.ac.kr
K. S. Dae, J. H. Jang
Electron Microscopy Research Group, Korea Basic Science Institute, Daejeon 34133, Korea
D.-Y. Cho
Department of Physics, Jeonbuk National University, Jeonju 54896, Korea
S. W. Lee
Department of Energy Systems Research and Department of Physics, Ajou University, Suwon 16499, Korea
E-mail: slee01@ajou.ac.kr

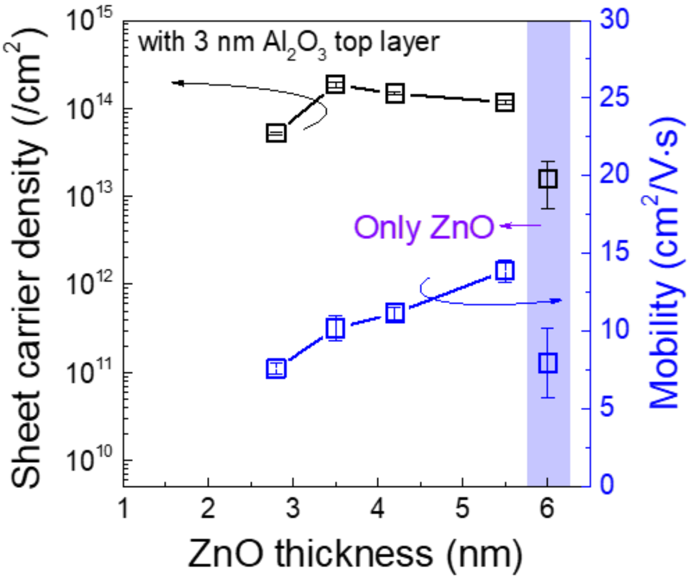

Figure S1. The sheet carrier density and Hall mobility for the 2DEG within the optimized ZnO thickness range (3~5.5 nm). For 5.5 nm-thick ZnO lower layer, the sheet carrier density is ~10^{14} cm^{-2} and Hall mobility is ~15 cm^2 V^{-1} s^{-1}. The purple box is data of only ZnO without Al_2O_3 upper layer.

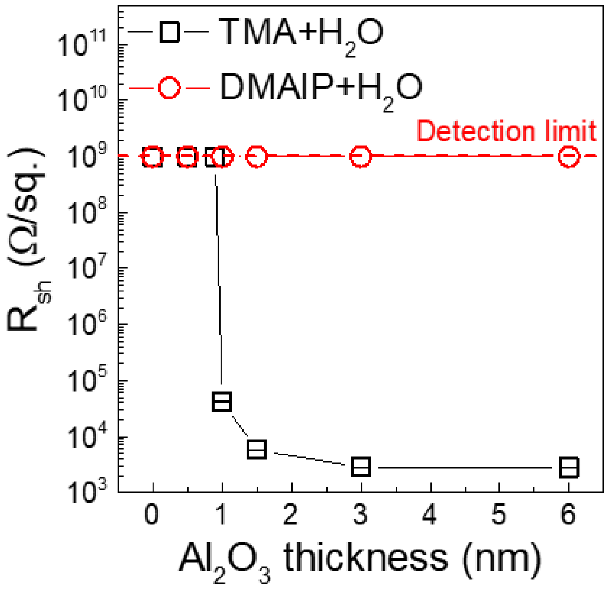

Figure S2. The effect of the surface reduction reaction on 2DEG formation. Sheet resistance of Al_2O_3/ZnO heterostructures as a function of Al_2O_3 thickness on 5.5 nm-thick ZnO lower layer, using a different Al precursor, dimethylaluminum isopropoxide (DMAIP), which has lesser reducing strength than TMA. The deposition temperature for both Al_2O_3 is 300 °C. Unlike TMA+H_2O process, showing abrupt resistance change at the critical thickness (1~1.5 nm), there is no change in the sheet resistance regardless of the Al_2O_3 thickness for DMAIP+H_2O process. This proves the conductivity of Al_2O_3/ZnO heterostructure is attributed to the surface reduction reaction, not Al doping in ZnO.

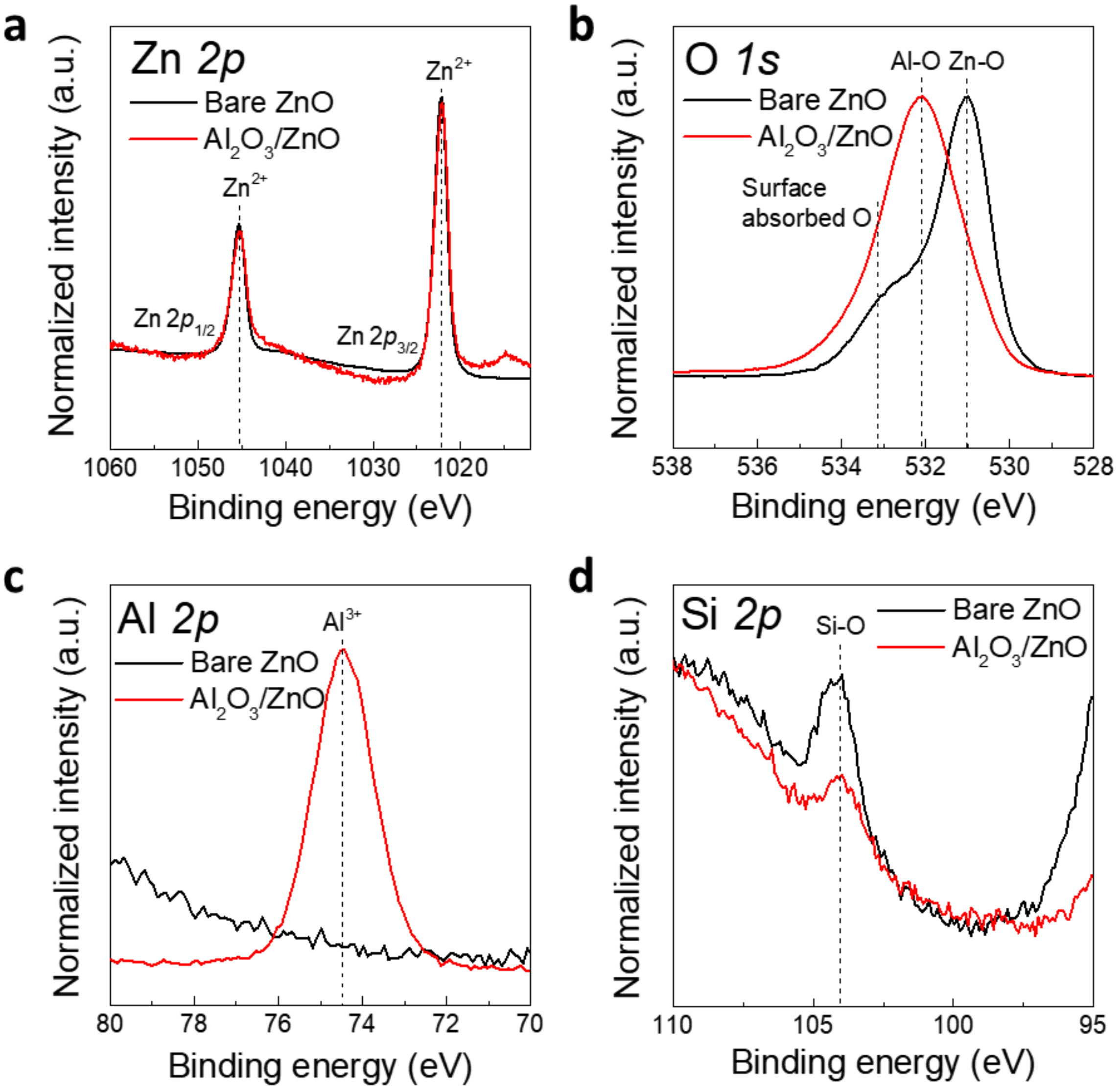

Figure S3. Chemical binding states of the bare ZnO and the Al_2O_3/ZnO heterostructure. Normalized XPS core level spectra of **a)** Zn *2p*, **b)** O *1s*, **c)** Al *2p*, and **d)** Si *2p* for bare ZnO (5.5 nm) and Al_2O_3 (3 nm)/ZnO (5.5 nm) heterostructure.

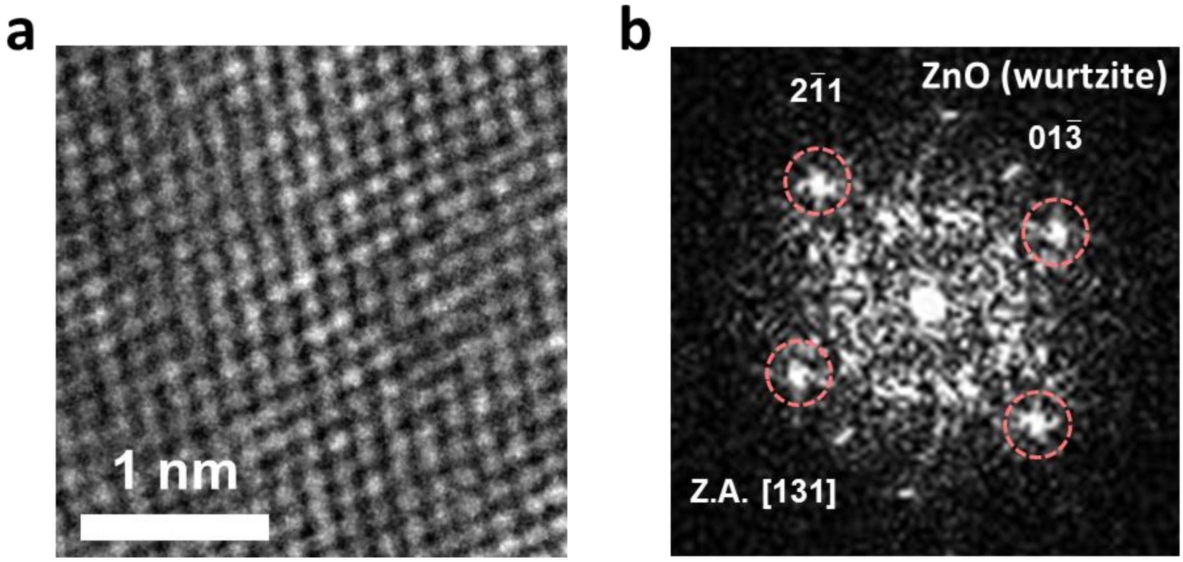

Figure S4. HRTEM analysis of the Al_2O_3/ZnO heterostructure. **a)** A magnified image of lattice fringe of 5.5 nm-thick ZnO and **b)** the corresponding diffraction pattern from FFT analysis. The (2-11) and (01-3) planes along the zone axis of [131] confirm the crystal structure of ZnO is hexagonal wurtzite.

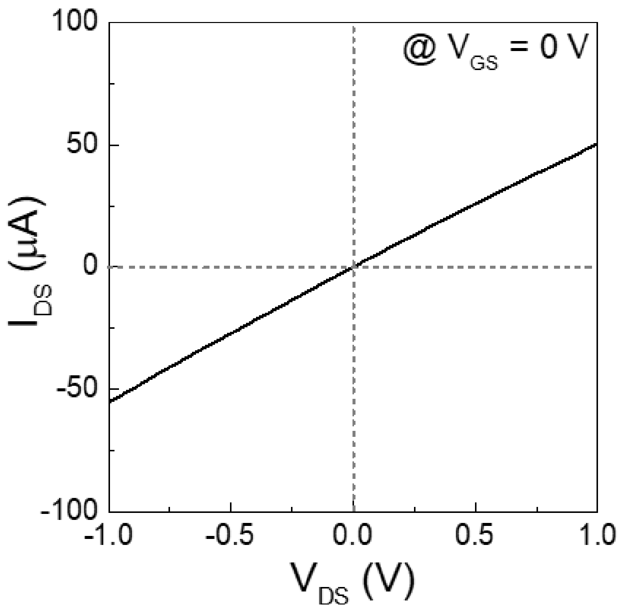

Figure S5. I_{DS}-V_{DS} curve of the 2DEG-FETs at the V_{GS} of 0 V. The I_{DS} was measured under both positive and negative bias polarities in the low V_{DS} range. The linearity of I_{DS}-V_{DS} curve confirms the ohmic behavior of S/D contact in the 2DEG-FETs.

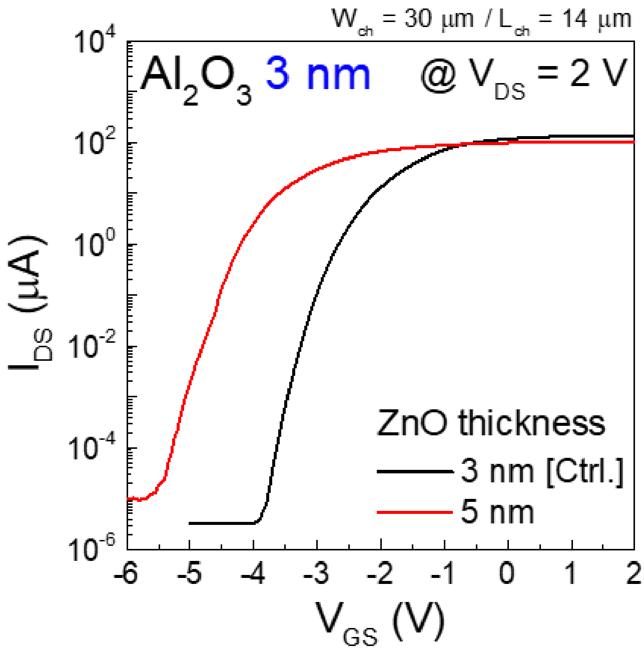

Figure S6. Transfer curves of the 2DEG-FETs with 3 nm- and 5 nm-thick ZnO layer. Even though the ZnO thickness decreases, the on current (I_{on}) maintained. This is because the I_{on} depends on the conductivity of the interface 2DEG regardless of ZnO thickness.

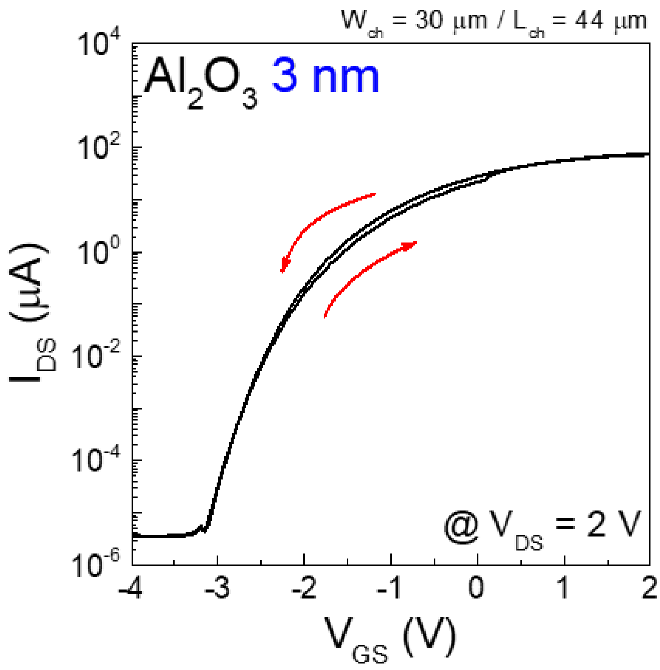

Figure S7. Transfer curve with hysteresis of the 2DEG-FETs. The hysteresis is negligible, indicating that electron trapping hardly occur within the operating voltage range.

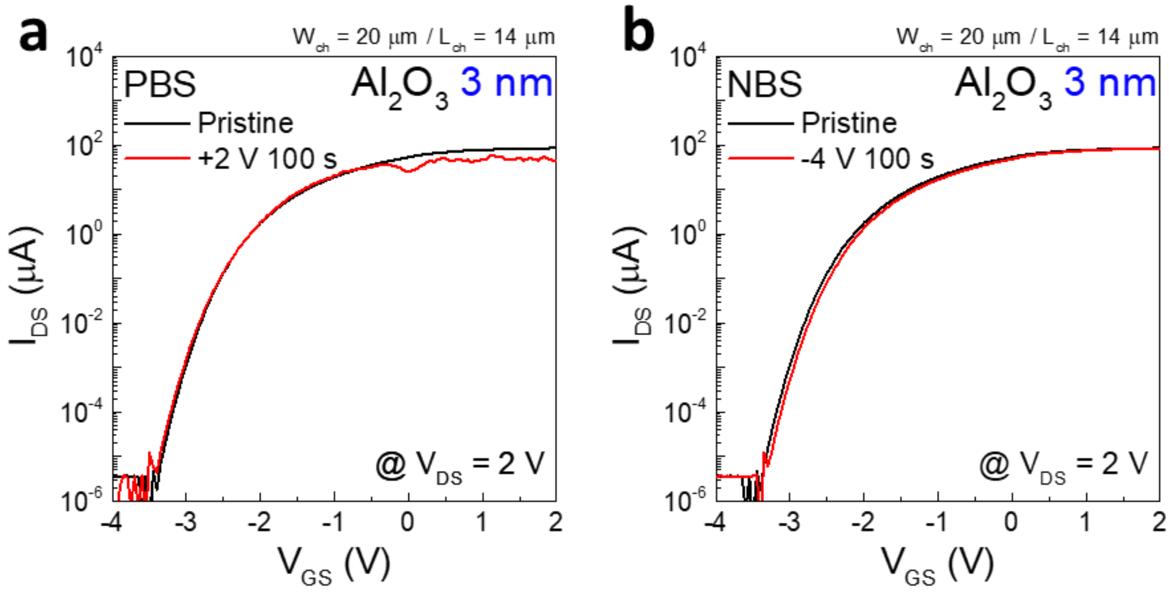

Figure S8. Stability of the 2DEG-FETs under applied gate bias stress. **a)** Transfer curve under positive bias stress (PBS) of +2 V for 100 s and **b)** under negative bias stress (NBS) of -4 V for 100 s, representing the maximum voltages for each bias polarity within the operating range. In both cases, noticeable shift was barely observed, indicating that electron trapping during device operation was negligible.

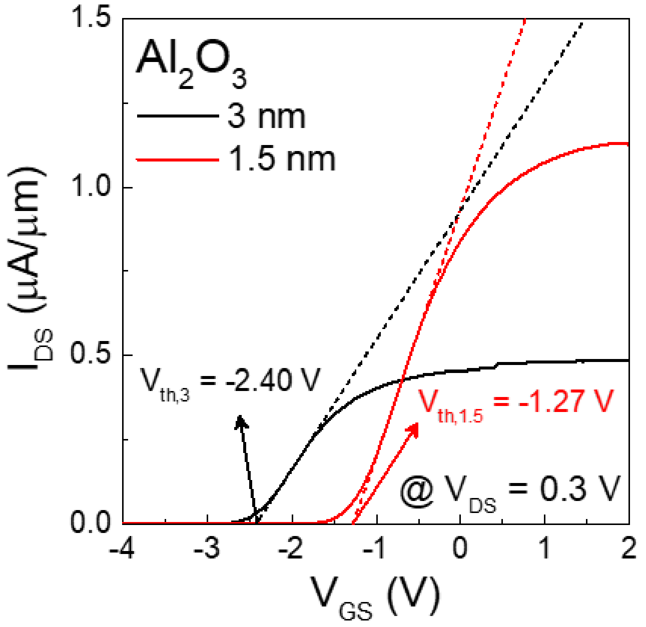

Figure S9. Threshold voltage of the 2DEG-FETs. Transfer curves of 3 nm and 1.5 nm 2DEG-FETs in linear scale at V_{DS} = 0.3 V. The V_{th} for each device was confirmed based on the extrapolation in the linear region method (ELR) using I_{DS} equation in the triode region. As the Al_2O_3 thickness decreases, the voltage drop in the film (V_{Al2O3}) is also reduced, resulting $|V_{th}|$ decrease with a distinct shift in the positive direction.

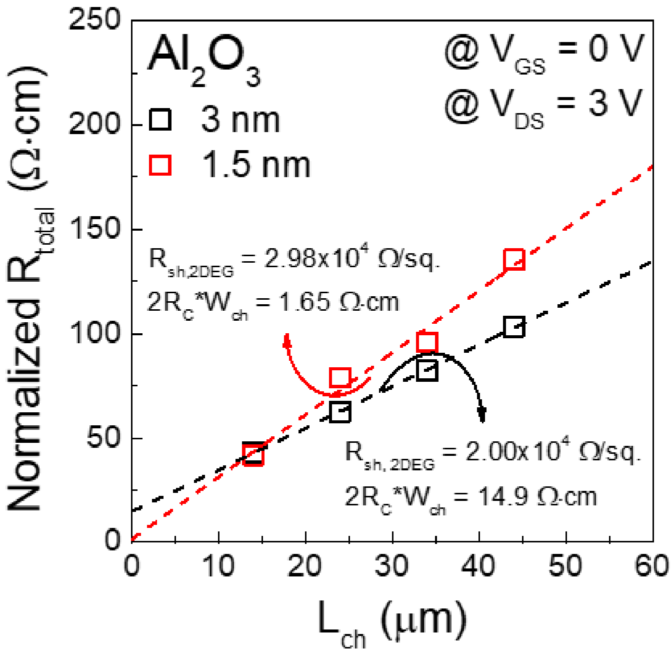

Figure S10. Transmission line measurement (TLM) in 2DEG-FETs. Resistances of 3 nm and 1.5 nm 2DEG-FETs were normalized with channel width (W_{ch}) as a function of channel length (L_{ch}). Total resistance consists of the resistance of 2DEG channel (R_{2DEG}) and two contact resistance ($2R_C$); $R_{total} = R_{2DEG} + 2R_C = R_{sh,2DEG}(L_{ch}/W_{ch}) + 2R_C$. By normalizing channel width, $R_{total}\times W_{ch} = R_{sh,2DEG}(L_{ch}) + 2R_C\times W_{ch}$, the slope and the y-intercept mean $R_{sh,2DEG}$ and $2R_C\times W_{ch}$, respectively.

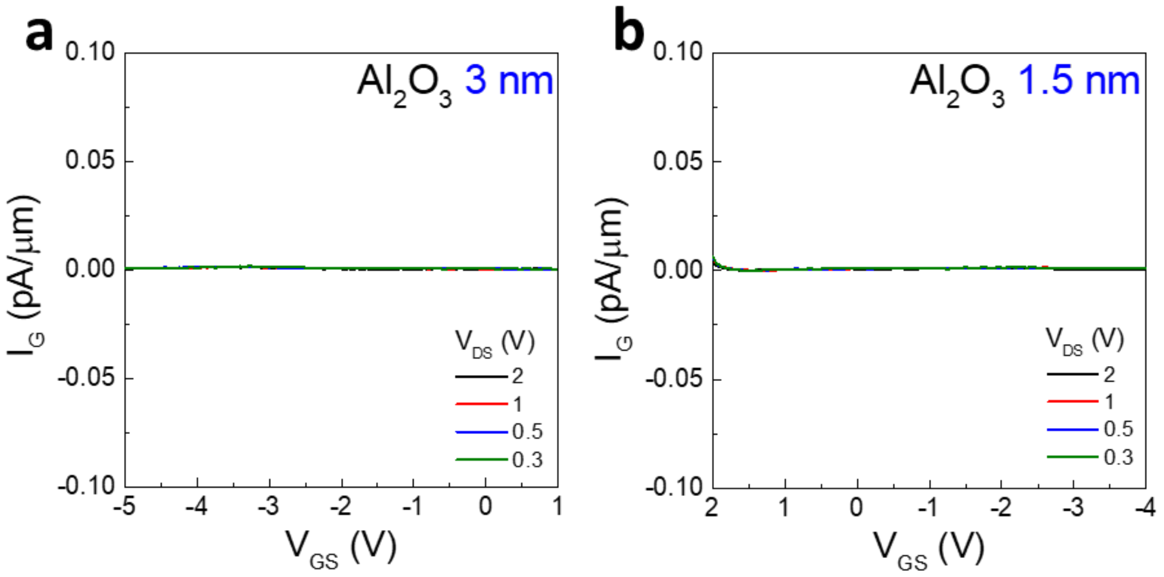

Figure S11. Gate leakage current (I_G) of the 2DEG-FETs. I_G as a function of V_{GS} for **a)** 3, and **b)** 1.5 nm 2DEG-FETs.

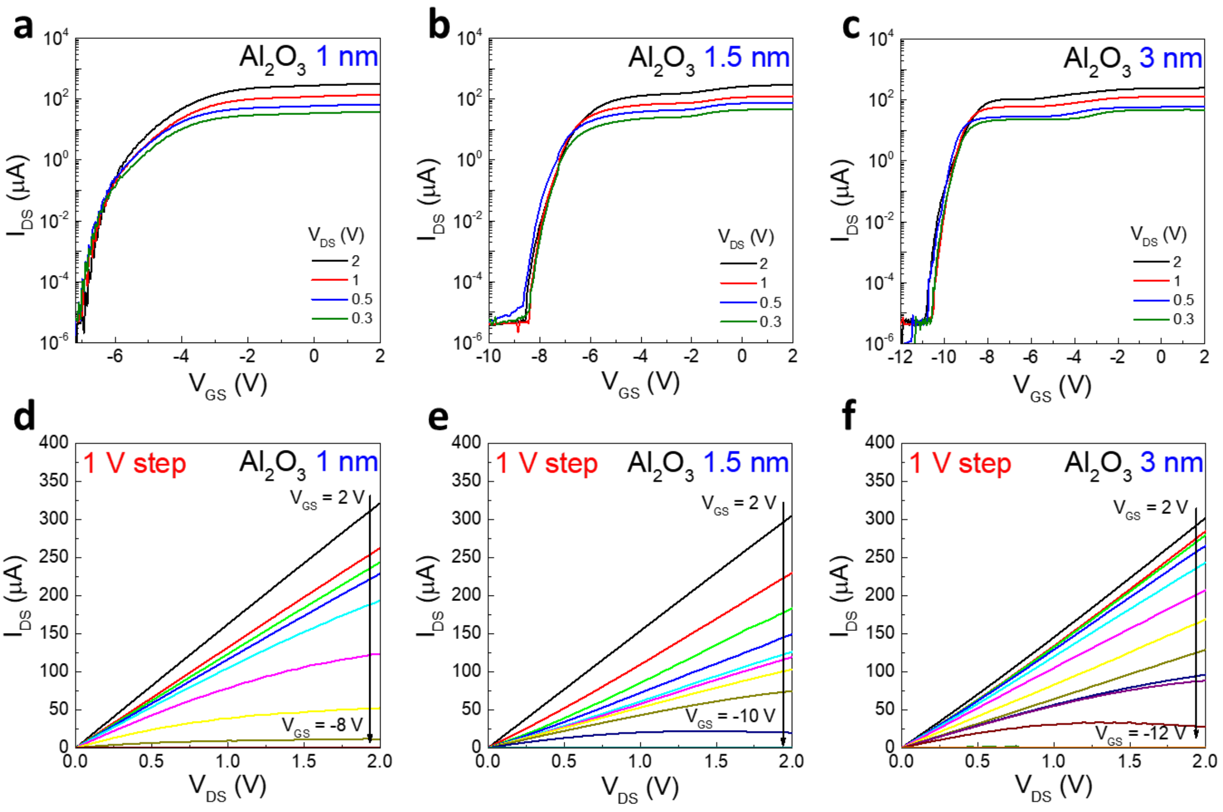

Figure S12. Transfer and output curves of the double-stacked 2DEG-FETs. Transfer curves of double-stacked 2DEG-FETs with **a)** 1, **b)** 1.5, and **c)** 3 nm-thick Al_2O_3 layer in the lower stack in log scale. Output curves of double-stacked 2DEG-FETs with **d)** 1, **e)** 1.5, and **f)** 3 nm-thick Al_2O_3 layer in the lower stack.

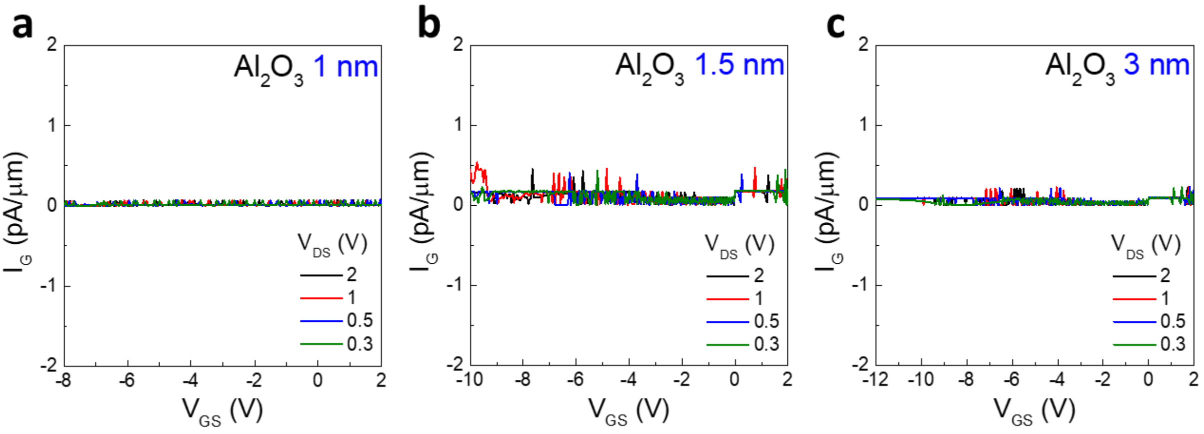

Figure S13. Gate leakage current (I_G) of the double-stacked 2DEG-FETs. I_G as a function of V_{GS} for double-stacked 2DEG-FETs with **a)** 1, **b)** 1.5, and **c)** 3 nm-thick Al_2O_3 layer in the lower stack.

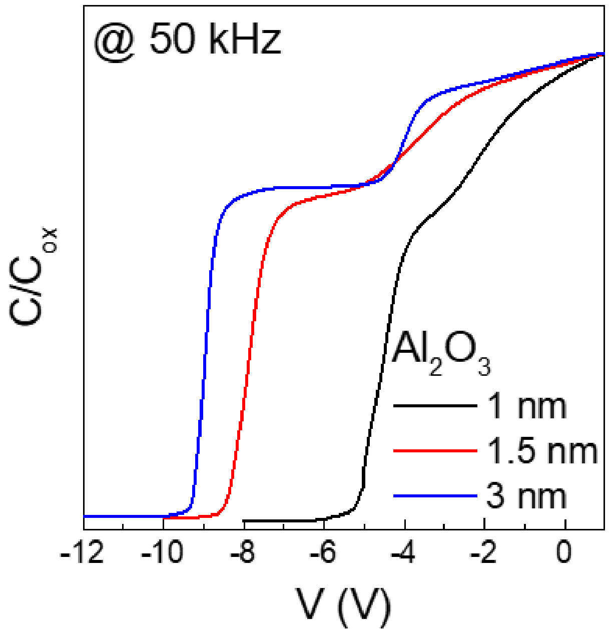

Figure S14. Capacitance-voltage curves of the double-stacked 2DEG-FETs with 1 nm-, 1.5 nm-, and 3 nm-thick Al_2O_3 layer in the lower stack, which were measured with the frequency of 50 kHz. The distinction of the intermediate state becomes more pronounced with increasing Al_2O_3 thickness, consistent with the transfer curves in Figure 3c-e.

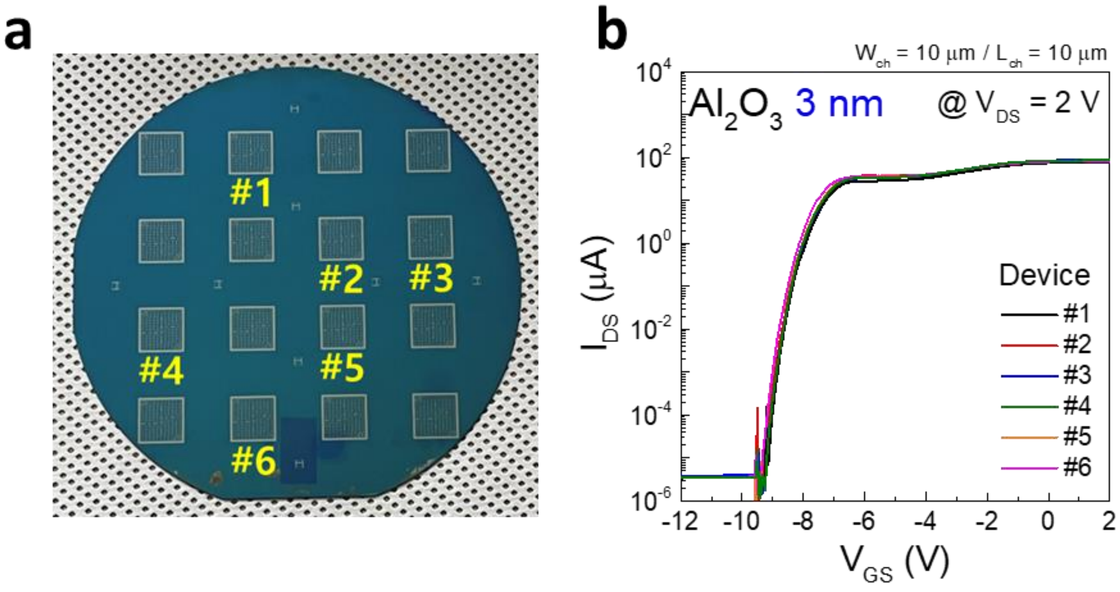

Figure S15. Device-to-device variation of double-stacked 2DEG-FETs with 3 nm-thick Al_2O_3 layer in the lower stack. **a)** An image of the devices integrated on a 4-inch wafer. **b)** Transfer curves of the devices integrated at each die.

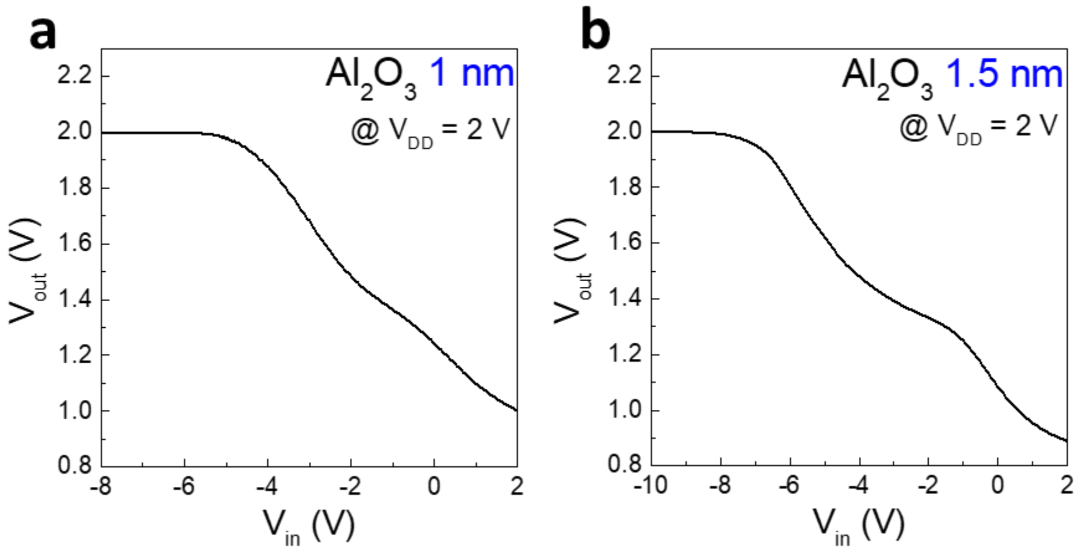

Figure S16. Voltage transfer curves (VTC) of the double-stacked 2DEG-FETs. The VTC of resistive-load ternary NMOS consisting of double-stacked 2DEG-FETs with **a)** 1 and **b)** 1.5 nm-thick Al_2O_3 layer in the lower stack. The operating voltage (V_{DD}) was fixed at 2 V. For the 1 nm case, due to the small V_{Al2O3}, the difference in V_{th} between stacked channels is not sufficient to allow each channel operate separately; thus, the intermediate state is hardly achieved. In contrast, for the 1.5 nm case, it appears that there are three states represented two curves due to slightly increased V_{Al2O3}, although they are not as distinct compared to the 3 nm case shown in Figure 3f. Both curves correspond directly to their I-V transfer curves in Figure 3c,d.

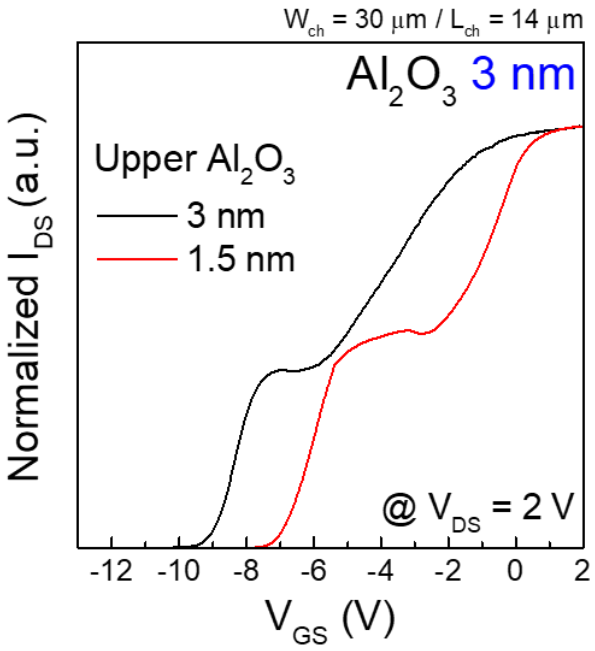

Figure S17. Normalized transfer curves of the double-stacked 2DEG-FETs with 1.5 nm- and 3 nm-thick Al_2O_3 in the upper stack and 3 nm-thick Al_2O_3 in the lower stack. The intermediate state range is wider when the thickness of the Al_2O_3 layer in the upper stack is reduced to 1.5 nm. This indicates that adjusting the thickness of both the upper and lower Al_2O_3 layers further expands the intermediate state range as the $|V_{th}|$ discrepancy of the stacked 2DEG channels increases.
